# P88 Interventions to address antimicrobial resistance in migrant populations: a systematic review

**DOI:** 10.1093/jacamr/dlaf230.095

**Published:** 2025-12-04

**Authors:** Lucy Lammie, Amelia Williams-Walker, Laura B Nellums, Catrin E Moore

**Affiliations:** Institute of Infection and Immunity, St George’s School of Health and Medical Sciences, City St George’s University, London, UK; Institute of Infection and Immunity, St George’s School of Health and Medical Sciences, City St George’s University, London, UK; College of Population Health, University of New Mexico, Albuquerque, NM, USA; Institute of Infection and Immunity, St George’s School of Health and Medical Sciences, City St George’s University, London, UK

## Abstract

**Background:**

Antimicrobial resistance (AMR) is a significant global health threat, disproportionately affecting structurally marginalized populations, including migrants, defined here as individuals living outside their country of birth. However, migrants are largely absent from global AMR surveillance and response strategies. This omission not only creates health inequities but also undermines global containment efforts due to the cross-border spread of resistant pathogens. This review draws on a systematic search to explore the existing evidence base for interventions targeting AMR in migrant populations.

**Objectives:**

Our primary objective was to summarize the current state of research on AMR interventions in migrant populations. This included identifying key gaps in data collection, intervention design and outcome measurement. A secondary objective was to propose a path forward for developing more equitable and effective AMR responses that are inclusive of migrant communities.

**Methods:**

We conducted a systematic search across three databases (MEDLINE, Embase and PubMed) for studies published globally between January 2000 and May 2025. The search was guided by PRISMA guidelines and combined search terms related to migration, AMR and interventions. We excluded studies on TB and focused on interventions addressing WHO-designated Critical and High Priority pathogens. A narrative synthesis was performed due to the limited number of eligible studies.

**Results:**

Our systematic search found 3861 records. Following our inclusion and exclusion criteria, only four eligible studies were identified, highlighting a significant lack of research in this area. Our narrative synthesis identified three persistent gaps. Firstly, data collection and surveillance are hampered by a lack of data that is stratified by migration status, ethnicity or country of origin in routine surveillance systems. This omission makes it difficult to understand the true scale of AMR in migrant populations and allocate necessary resources accordingly. Secondly, intervention design in the papers focused on hospital-based approaches which failed to account for the unique barriers migrants face, including precarious housing, frequent relocation and restricted healthcare access. Such factors limit the reach and effectiveness of these strategies. Finally, we found a lack of robust outcome measurements. Studies predominantly reported microbiological outcomes (e.g. transmission events) rather than patient-centred, clinical or long-term health effects. This focus on preventing transmission to the host population, rather than on the health outcomes of migrants themselves, reflects a broader systemic bias and provides only limited guidance for developing truly equitable strategies.

**Conclusions:**

Current evidence on AMR interventions for migrant populations is scarce and fragmented, reflecting a systemic gap in public health planning globally. To address these deficiencies, future efforts must prioritize the collection of high-quality, disaggregated surveillance data. They should also promote the development of contextually appropriate, community-based care models and ensure robust, equity-driven evaluation of interventions. Addressing these gaps is crucial not only for protecting migrant health, but also for strengthening health systems and achieving sustainable global progress against AMR.

